# Antimicrobial resistance profile of methicillin-resistant *Staphylococcus aureus* isolates in children reported from the ISPED surveillance of bacterial resistance, 2016–2021

**DOI:** 10.3389/fcimb.2023.1102779

**Published:** 2023-01-19

**Authors:** Xia Wu, Chuanqing Wang, Leiyan He, Hongmei Xu, Chunmei Jing, Yinghu Chen, Aiwei Lin, Jikui Deng, Qing Cao, Huiling Deng, Huijun Cai, Yiping Chen, Jinhong Yang, Ting Zhang, Yuanyuan Huang, Jianhua Hao, Hui Yu

**Affiliations:** ^1^ Department of Infectious Diseases, Children’s Hospital of Fudan University, Shanghai, China; ^2^ Department of Clinical Laboratory, Children’s Hospital of Fudan University, Shanghai, China; ^3^ Department of Infectious Diseases, Children’s Hospital of Chongqing Medical University, Chongqing, China; ^4^ Department of Clinical Laboratory, Children’s Hospital of Chongqing Medical University, Chongqing, China; ^5^ Department of Infectious Diseases, Children’s Hospital of Zhejiang University School of Medicine, Hangzhou, China; ^6^ Department of Infectious Diseases, Qilu Children’s Hospital of Shandong University, Jinan, China; ^7^ Department of Infectious Diseases, Shenzhen Children’s Hospital, Shenzhen, China; ^8^ Department of Infectious Diseases, Shanghai Children’s Medical Center of Shanghai Jiaotong University School of Medicine, Shanghai, China; ^9^ Department of Infectious Diseases, Xi’an Children’s Hospital, Xi’an, China; ^10^ Department of Clinical Laboratory, Xi’an Children’s Hospital, Xi’an, China; ^11^ Department of Pediatric Infectious Diseases, Second Affiliated Hospital & Yuying Children’s Hospital of Wenzhou Medical University, Wenzhou, China; ^12^ Department of Clinical Laboratory, Second Affiliated Hospital & Yuying Children’s Hospital of Wenzhou Medical University, Wenzhou, China; ^13^ Department of Gastroenterology and Infectious Diseases, Children’s Hospital of Shanghai Jiaotong University School of Medicine, Shanghai, China; ^14^ Department of Pediatrics, Bethune First Hospital of Jilin University, Changchun, China; ^15^ Department of Infectious Diseases, Kaifeng Children’s Hospital, Kaifeng, China

**Keywords:** methicillin-resistant *Staphylococcus aureus*, antimicrobial resistance, children, infectious disease surveillance of pediatrics (ISPED), neonates

## Abstract

**Introduction:**

Methicillin-resistant *Staphylococcus aureus* (MRSA) poses a serious threat to public health worldwide. In December 2015, the Infectious Disease Surveillance of Pediatrics (ISPED) program was organized to monitor bacterial epidemiology and resistance trends in children.

**Methods:**

This retrospective study was conducted from January 2016–December 2021 on patients at eleven ISPED-group hospitals.

**Results:**

From 2016–2021, a total of 13024 MRSA isolates were obtained from children. The most common age group for patients with MRSA infection was less than 3 years old, and newborns were an important group affected by MRSA infection. MRSA was most commonly isolated from the lower respiratory, an abscess, a secretion, or blood in neonates and from the lower respiratory, an abscess, or the upper respiratory in non-neonates. All isolates were susceptible to vancomycin and linezolid and resistant to penicillin; additionally, 76.88%, 54.97%, 22.30%, 5.67%, 5.14%, 3.63%, and 1.42% were resistant to erythromycin, clindamycin, tetracycline, levofloxacin, sulfamethoxazole-trimethoprim (TMP-SMX), gentamicin, and rifampin, respectively. Between 2016 and 2021, a significant increase was seen in the levofloxacin- and TMP-SMX-resistance rates (from 5.45% to 7.14% and from 4.67% to 6.50%, respectively) among MRSA isolates, along with a significant decrease in the rates of resistance to erythromycin (from 82.61% to 68.08%), clindamycin (from 60.95% to 46.82%), tetracycline (from 25.37% to 17.13%), gentamicin (from 4.53% to 2.82%), and rifampin (from 1.89% to 0.41%).

**Discussion:**

The antibiotic-resistance rates varied among MRSA isolated from different sources. Because of the high antibiotic resistance rate to clindamycin, this antibiotic is not recommended for empirical treatment of MRSA infections, especially in osteomyelitis.

## Introduction

Methicillin-resistant *Staphylococcus aureus* (MRSA), which was first identified in 1961, poses a serious threat to public health worldwide owing to its significant resistance to antibiotics. Among community-associated *S. aureus* infections in children, 63.6% were found to be due to MRSA (Wang et al., 2017). Previous studies in children found that MRSA was responsible for 45.8%–75.6% of *S. aureus* pneumonia cases (Doudoulakakis et al., 2017; Song et al., 2017), 44% of *S. aureus* bacteraemia cases (Kumarachandran et al., 2017), and 32.8% of cases with an *S. aureus* central nervous system infection (Vallejo et al., 2017). Recently, in a study of bloodstream infections in China, the percentage of cases due to MRSA in non-intensive care unit (ICU) patients increased significantly from 8.4% in 1998–2002 to 68.3% in 2013–2017 (Tian et al., 2019).

MRSA can cause invasive, life-threatening systemic infections, e.g., in severe sepsis and necrotizing pneumonia, which are particularly problematic in children (Liu et al., 2011; Lim et al., 2014). The high antibiotic resistance of MRSA is very concerning because it can lead to treatment failure in clinical practice. Currently, MRSA infections remain prevalent and account for significant morbidity and mortality worldwide. MRSA infections in children have been associated with a longer duration of bacteraemia, longer length of hospital stay, higher likelihood of complications, and greater mortality rate compared with methicillin-sensitive *S. aureus* (MSSA) infections (Burke et al., 2009; Hamdy et al., 2019).

Given the heavy burden associated with MRSA infections, there is an urgent need to understand the distribution and antimicrobial susceptibilities of MRSA. Here, to investigate the profiles of MRSA infection and MRSA drug resistance in children, we compared the distribution and antimicrobial susceptibilities of MRSA isolates in cases from eleven hospitals within the Infectious Diseases Surveillance of Pediatrics (ISPED) group of China over a six-year period (2016–2021).

## Patients and methods

### Surveillance population

This retrospective study was conducted across eleven hospitals within the ISPED group of China from January 2016 to December 2021. We reviewed the medical records of patients who were younger than 18 years and had any clinical culture that yielded an isolate of MRSA. We collected the following data from the patient medical records: sex, age, infection site, and antibiotic resistance profile. Our analysis included a total of 13024 MRSA clinical isolates obtained from the following eleven hospitals: Children’s Hospital of Chongqing Medical University, Children’s Hospital of Fudan University, Children’s Hospital of Zhejiang University School of Medicine, Qilu Children’s Hospital of Shandong University, Shenzhen Children’s Hospital, Shanghai Children’s Medical Center of Shanghai Jiaotong University School of Medicine, Xi’an Children’s Hospital, Second Affiliated Hospital & Yuying Children’s Hospital of Wenzhou Medical University, Children’s Hospital of Shanghai Jiaotong University School of Medicine, Bethune First Hospital of Jilin University, and Kaifeng Children’s Hospital.

### Antimicrobial susceptibility testing (AST)

The antibiotic susceptibility testing was performed in each participating site. Antimicrobial susceptibility testing (AST) was performed using the Kirby-Bauer method or automated systems. AST was conducted for penicillin, oxacillin, erythromycin, clindamycin, levofloxacin, sulfamethoxazole-trimethoprim (TMP-SMX), gentamicin, rifampin, and minocycline. MRSA were identified based on their resistance to oxacillin. The standard strains ATCC 25922, ATCC 29213, and ATCC 29212 were used as quality-control strains for the antimicrobial susceptibility tests. The AST breakpoint criteria of the Clinical and Laboratory Standards Institute (CLSI) were adopted (Clinical and Laboratory Standards Institute, 2019).

### Statistical analysis

Statistical significance was calculated by applying the χ^2^ test, or the Fisher’s exact test in the case of small sample sizes, using the SPSS (Version 20) statistics program. Statistical significance in this study was defined as a *P*-value of < 0.05.

## Results

### Distribution of MRSA

From 2016 to 2021, the yearly total number of *S. aureus* isolates obtained ranged from 4231 to 8561. During these years, the respective proportions of *S. aureus* isolates that were MRSA isolates were 31.50%, 36.80%, 34.10%, 34.41%, 35.00%, and 32.31%. A total of 13024 MRSA isolates, of which 6.10% (794) were collected from outpatients and 93.90% (12230) were collected from inpatients, were obtained from children in this study. The distribution of MRSA in inpatients was distinct from that in outpatients; MRSA isolates were detected much more commonly from inpatients than from outpatients (34.85% vs. 25.56%, χ^2^ = 109.564, *P* = 0.000).

**Figure 1:**
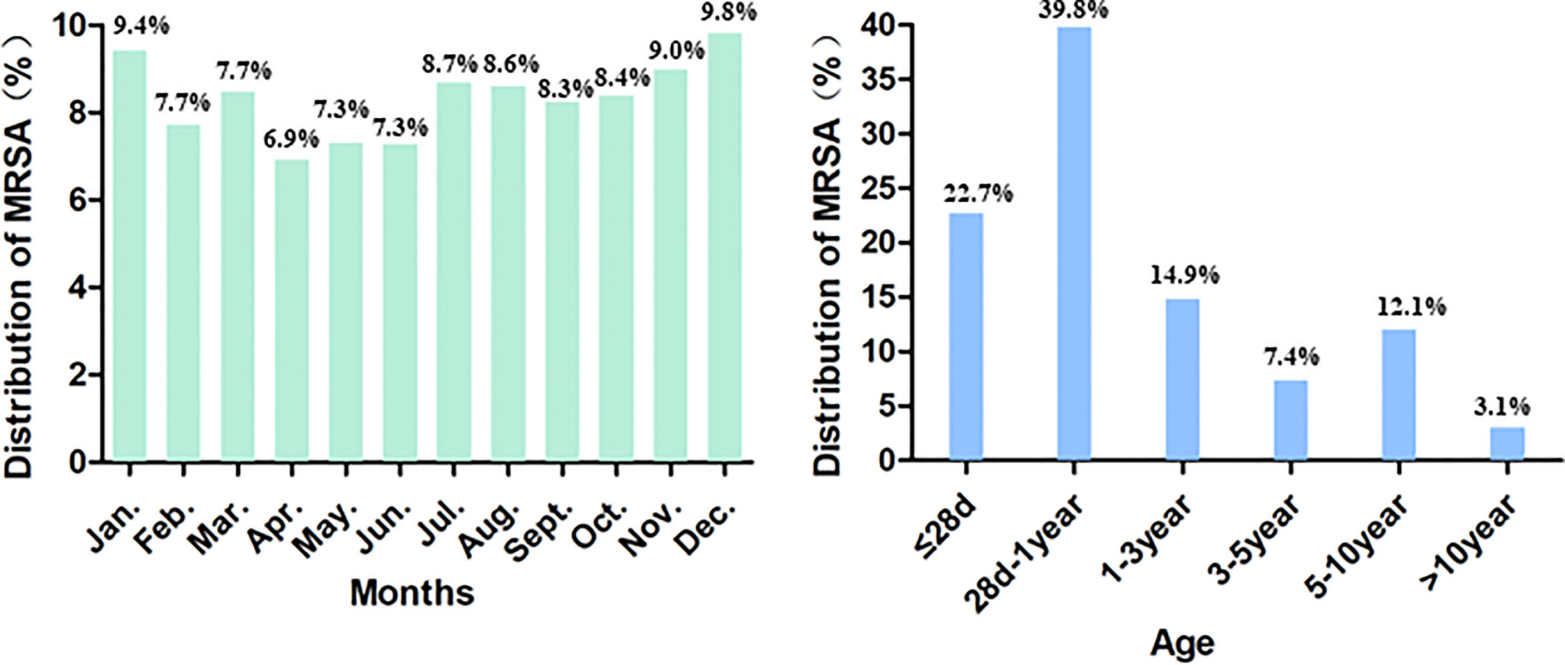
Demographics of patients with MRSA infections.

Among the included 13024 cases, 7763 (59.61%) of the patients were male. The median patient age was 5 months (range: 1 day to 17 years), and 2902 (22.73%) of the patients were newborn infants (aged ≤28 days). MRSA cases were detected much more commonly during the months of January, November, and December (**Figure 1**).

MRSA was most commonly isolated from the lower respiratory (58.28%), followed by from an abscess (16.78%), a secretion (7.61%), blood (4.64%), the upper respiratory (4.45%), urine (0.98%), a bone or joint (0.27%), the central nervous system (0.24%), and other sources. Notably, compared with those in non-neonates, the constituent proportions of MRSA isolates obtained from blood, secretions, and urine were higher, while those obtained from respiratory sites and bone or joint sites were lower, in neonates (**Table 1**).

**Table 1:** Distributions of MRSA in neonates and nonneonates in 2016 to 2021.

Specimen type	Neonates % (n=2960)	Nonneonates % (n=10064)	χ^2^	P-Value
Lower respiratory	48.55%	61.73%	160.180	0.000
Upper respiratory	1.97%	5.27%	56.281	0.000
Blood	6.19%	4.16%	20.888	0.000
Abscess	18.00%	16.53%	3.406	0.065
Secretion*	13.56%	5.96%	174.783	0.000
Urine	1.49%	0.84%	9.493	0.002
bone and joint	0.07%	0.30%	4.047	0.032
Cerebrospinal fluid	0.10%	0.28%	2.301	0.089
Other	10.07%	4.92%	–	–

*Secretion: Umbilical secretion, wound.

### Bacterial identification and AST

**Figure 2:**
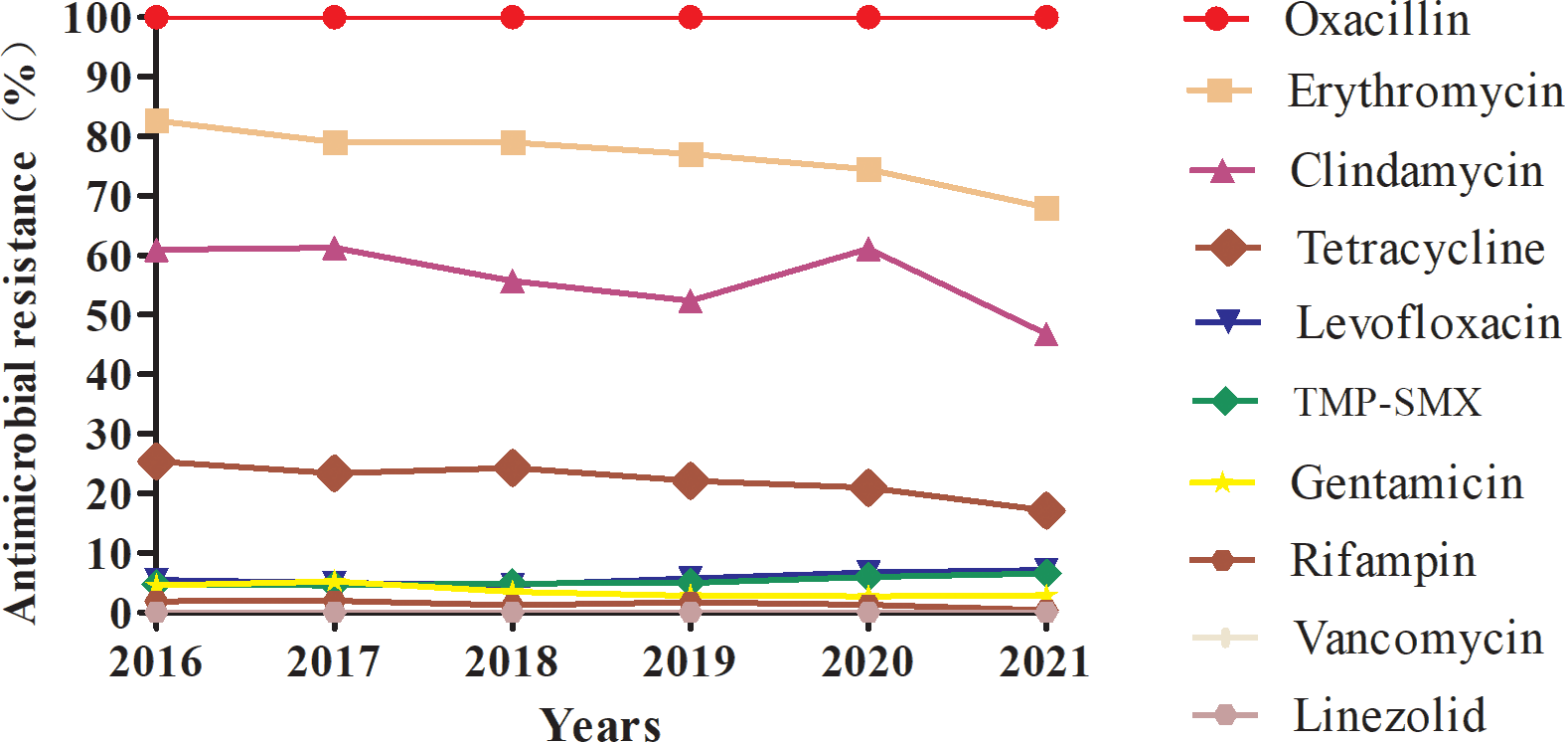
Profile of resistance to ten main antimicrobials (%) of MRSA isolates collected from 2016 to 2021.

The antimicrobial resistance profiles of the 13024 MRSA isolates from this study are provided in **Table 2** and **Table 3**. All study isolates were susceptible to vancomycin and linezolid and were resistant to penicillin; additionally, 76.88% of the study isolates were resistant to erythromycin, 54.97% to clindamycin, 22.3% to tetracycline, 5.67% to levofloxacin, 5.14% to TMP-SMX, 3.63% to gentamicin, and 1.42% to rifampin. The rifampin-resistance rate of MRSA isolates derived from inpatients was significantly higher than those of MRSA isolates derived from outpatients (1.51% vs. 0.13%, χ2 = 10.119, P = 0.001), while tetracycline-resistance rate of inpatients-derived MRSA isolates was lower than that of outpatients-derived strains (22.00% vs. 28.57%, χ2 = 11.111, P = 0.001). Changes in the antibiotic resistance rates among the MRSA isolates from 2016 to 2021 were observed, with an increase in the rates of resistance to levofloxacin (from 5.45% to 7.14%) and TMP-SMX (from 4.67% to 6.50%) and a decrease in the rates of resistance to erythromycin (from 82.61% to 68.08%), clindamycin (from 60.95% to 46.82%), tetracycline (from 25.37% to 17.13%), gentamicin (from 4.53% to 2.82%), and rifampin (from 1.89% to 0.41%) (**Table 3**, **Figure 2**).

**Table 2:** Antimicrobial resistance rates of MRSA strains in inpatients and outpatients.

Antibiotic	Inpatients (n=12230)	Outpatients (n=794)	Total (n=13024)	χ2	P-Value
Penicillin	100.00	100.00	100.00	–	–
Oxacillin	100.00	100.00	100.00	–	–
Erythromycin	76.84	77.41	76.88	0.134	0.714
Clindamycin	54.97	54.87	54.97	0.003	0.954
Tetracycline	22.00	28.57	22.30	11.111	0.001
Levofloxacin	5.78	4.09	5.67	3.775	0.052
TMP-SMX	5.06	6.46	5.14	3.014	0.083
Gentamicin	3.63	3.65	3.63	0.001	0.977
Rifampin	1.51	0.13	1.42	10.119	0.001
Vancomycin	0.00	0.00	0.00	–	–
Linezolid	0.00	0.00	0.00	–	–

**Table 3:** Antimicrobial resistance rates of MRSA strains in 2016 to 2021.

Antibiotic	2016 % (n=2100)	2017 % (n=2258)	2018 % (n=2241)	2019 % (n=2946)	2020 % (n=1481)	2021 % (n=2000)	χ^2^	P-Value
Penicillin	100.00	100.00	100.00	100.00	100.00	100.00	–	–
Oxacillin	100.00	100.00	100.00	100.00	100.00	100.00	–	–
Erythromycin	82.61	79.03	78.98	77.01	74.44	68.08	135.964	0.000
Clindamycin	60.95	61.28	55.69	52.37	61.10	46.82	137.725	0.000
Tetracycline	25.37	23.39	24.33	22.11	20.94	17.13	30.224	0.000
Levofloxacin	5.45	4.98	4.66	5.71	6.74	7.14	16.114	0.007
TMP-SMX	4.67	4.51	4.87	4.96	5.97	6.50	12.242	0.032
Gentamicin	4.53	5.22	3.48	2.81	2.68	2.82	30.908	0000
Rifampin	1.89	1.94	1.21	1.73	1.29	0.41	21.895	0.000
Vancomycin	0.00	0.00	0.00	0.00	0.00	0.00	–	–
Linezolid	0.00	0.00	0.00	0.00	0.00	0.00	–	–

The activity of the tested antimicrobials against the 13024 MRSA isolates obtained from different infection sites is summarized in **Table 4**. The erythromycin-resistance rates of blood-derived (79.62%) and abscess-derived (79.29%) MRSA isolates were significantly higher than that of urine-derived isolates (70.87%; χ^2^ = 4.645 and 5.089, *P* = 0.031 and 0.024, respectively). The clindamycin-resistance rates of bone and joint-derived (72.73%) and urine-derived (63.49%) MRSA isolates were significantly higher than that of lower respiratory-derived isolates (54.61%; χ^2^ = 4.351 and 3.942, *P* = 0.037 and 0.047, respectively). The tetracycline-resistance rate of bone and joint-derived MRSA isolates was significantly higher than those of MRSA isolates derived from other sources (50.00% vs. 20.42%–31.82%, χ^2^ = 63.809, *P* = 0.000). The levofloxacin-resistance rate of blood-derived MRSA isolates (8.07%) was significantly higher than those of abscess-derived (4.48%) and secretion-derived (5.15%) isolates (χ^2^ = 11.548 and 5.098, *P* = 0.001 and 0.024, respectively). The TMP-SMX-resistance rates of secretion-derived (6.15%) and blood-derived (6.12%) MRSA isolates were significantly higher than that of upper respiratory-derived isolates (3.50%; χ^2^ = 5.144 and 4.352, *P* = 0.023 and 0.037, respectively).

**Table 4:** Antibiotic resistance rates of MRSA from different site of infection.

Antibiotic	Upper respiratory % (n=579)	Lower respiratory % (n=7590)	Blood %(n=604)	Abscess %(n=2185)	Secretion %(n=991)	Urine %(n=128)	bone and joint %(n=35)	Cerebrospinal fluid %(n=31)	χ^2^	P-Value
Penicillin	100.00	100.00	100.00	100.00	100.00	100.00	100.00	100.00	–	–
Oxacillin	100.00	100.00	100.00	100.00	100.00	100.00	100.00	100.00	–	–
Erythromycin	78.37	76.71	79.62	79.29	76.19	70.87	78.79	80.65	12.231	0.093
Clindamycin	58.16	54.61	57.67	58.70	59.77	63.49	72.73	54.84	25.464	0.001
Tetracycline	22.43	20.42	28.61	28.13	23.21	25.00	50.00	31.82	63.789	0.000
Levofloxacin	5.72	6.07	8.07	4.48	5.15	7.94	0.00	3.23	17.064	0.017
TMP-SMX	3.50	4.75	6.12	5.87	6.15	7.09	8.57	3.45	12.961	0.073
Gentamicin	4.63	3.79	3.70	3.06	3.11	3.94	2.86	0.00	6.154	0.522
Rifampin	0.87	1.60	1.58	1.06	2.12	0.79	0.00	0.00	8.667	0.277
Vancomycin	0.00	0.00	0.00	0.00	0.00	0.00	0.00	0.00	–	–
Linezolid	0.00	0.00	0.00	0.00	0.00	0.00	0.00	0.00	–	–

MRSA, methicillin-resistant Staphylococcus aureus; TMP-SMX, sulfamethoxazole-trimethoprim.

## Discussion

We observed the antimicrobial resistance trends of MRSA isolates obtained from children participating in ISPED over the six-year period from 2016 to 2021. Despite the reduction in the proportion of MRSA among *S. aureus* infections that was noted over the recent 10-year span (Kramer et al., 2019), the incidence of MRSA has remained high in some areas (Takemura and Mochizuki, 2018), and MRSA remains a predominant cause of infection. The proportions of MRSA among *S. aureus* infections in adults, according to the China Antimicrobial Surveillance Network (CHINET) report, decreased from 69.94% in 2005 to 31.00% in 2020 (Hu et al., 2016; Hu et al., 2017; Hu et al., 2018; Hu et al., 2020a; Hu et al., 2020b; Hu et al., 2021). In contrast, the proportions of MRSA among paediatric *S. aureus* infections found in our study remained in the range of 31.50%–36.80% over the past six years. Here, MRSA cases were detected much more commonly in the months of January, November, and December, during which the weather in China becomes colder. The seasonal nature of MRSA infections varied based on specimen source, with wound infections more prevalent in warmer months, and respiratory infections more prevalent during colder months (Delorme et al., 2017; Marcelin et al., 2017). The most common age group of patients with MRSA infection is <5 years old (Perovic et al., 2015; Ilczyszyn et al., 2016). In the present study, the median patient age was only 5 months old. The most common age stage of patients participating in our study was 28 d–1 year (39.8%), and 77.4% of study participants were aged less than 3 years old, which is younger compared with the participants studied in other reports (Milstone et al., 2010). In addition, 22.73% of the patients in our study were newborn infants, and newborns are also an important group affected by MRSA infections.

MRSA is a particularly threatening pathogen in children because it causes infections of multiple organ systems. Previous research has found that the most common sites of MRSA infection are the respiratory tract (36.3%–48.0%), followed by the blood (15%–35.7%), wound or intravenous sites (25.2%–44%), and the urinary tract (2.8%–28%), while the least common source of MRSA isolates is puncture fluid (containing hydrothorax, ascites, pericardial fluid, cerebrospinal fluid, or articular cavity fluid; 21.32%) (Rahimi and Shokoohizadeh, 2016; Huang et al., 2019; Samadi et al., 2019). MRSA is an important cause of bloodstream infections (BSI), and in 2009–2017, the MRSA detection rate was 40%–50% among BSI-associated *S. aureus* (Zhang et al., 2022). Additionally, *S. aureus* was reported to be the main pathogen (67.5%) in paediatric osteomyelitis, and the proportion of MRSA among the cases due to *S. aureus* was 44% (Chen et al., 2021). In the present work, the most common clinical sources of MRSA infection were the respiratory tract (62.73%), followed by an abscess (16.78%), a secretion (7.61%), and blood (4.64%), and the constituent proportions isolated from blood, secretions, and urine were higher in neonates compared with those in non-neonates. An increasing incidence of MRSA infections was observed among neonates (Dolapo et al., 2014). Because immunologically immature children are more susceptible to MRSA infection, neonates are more likely to develop MRSA BSIs compared with non-neonates. In neonates with MRSA infection, the proportion of umbilical infection is high, and BSI may be associated with umbilical cord infection. Therefore, it is important to monitor the MRSA epidemiology and resistance trends in neonates.

MRSA antibiotic resistance has been reported in many countries. The significant antibiotic resistance of MRSA is a particular concern because it can lead to treatment failure in clinical practice. Several studies of MRSA antimicrobial susceptibility have revealed their high rates of resistance to erythromycin, clindamycin, levofloxacin, and ciprofloxacin (Decousser et al., 2018; Liang et al., 2019). Similarly, among MRSA isolates from paediatric patients, the highest percentage of isolates was resistant to erythromycin (62%), followed by those resistant to clindamycin (14%–57%), TMP-SMX (3%–24%), gentamicin (24%), rifampicin (12%), or minocycline (10%), while all isolates were susceptible to both vancomycin and linezolid (Milstone et al., 2010; Miguel et al., 2019). In the CHINET surveillance report of MRSA resistance trends between 2005 and 2020, decreases were observed in the percentages of isolates resistant to clindamycin (from 90.1% to 58.6%), erythromycin (from 92.7% to 78.9%), levofloxacin (from 83.3% to 32.6%), gentamicin (from 77.3% to 20.7%), rifampin (from 34.9% to 8.2%), and TMP-SMX (from 36.3% to 6.4%) (Hu et al., 2016; Hu et al., 2019; Hu et al., 2020; Hu et al., 2021). In the present study, our MRSA isolates had high rates of resistance to erythromycin and clindamycin but low rates of resistance to levofloxacin, TMP-SMX, gentamicin, and rifampin, and all these isolates were susceptible to vancomycin and linezolid. Changes in the antibiotic-resistance profile of MRSA isolates from 2016 to 2021 were noted, with an increase in the levofloxacin- and TMP-SMX-resistance rates (from 5.45% to 7.14% and from 4.67% to 6.50%, respectively), and a decrease in the erythromycin-, tetracycline-, gentamicin-, and rifampin-resistance rates. The decreased rates of resistance to these antibiotics may be related to their decreased use in recent years. In our monitoring, the clindamycin-resistance rate remained at 50%–60% from 2016 to 2020, and although this rate decreased to 47% in 2021, it remained close to 50%.

MRSA isolates obtained from different clinical sources may exhibit different levels of antimicrobial susceptibility. A study in Chicago suggested that MRSA isolates obtained from the blood are more likely to be drug resistant compared with MRSA skin and soft tissue isolates (Acree et al., 2017). Liang et al. reported that the rates of gentamicin and ciprofloxacin resistance were significantly higher in MRSA isolates derived from the respiratory tract than in those isolated from the skin and soft tissue or the blood (Liang et al., 2019). Resistance to antibiotics was prevalent among MRSA isolates cultured from patients with ocular infections at US centres, with 72.7% of isolates resistant to fluoroquinolones and 92.9% resistant to azithromycin (Asbell et al., 2020). We found that the erythromycin-resistance rates of blood- and abscess-derived MRSA isolates were higher than that of MRSA isolates obtained from other clinical sources. Additionally, the rates of clindamycin and tetracycline resistance among bone and joint-derived MRSA isolates were significantly higher than those obtained from other clinical sources.

An understanding of the distribution and antimicrobial susceptibilities of MRSA strains will be crucial for guiding antibiotic treatment in MRSA cases. Presently, vancomycin, linezolid, and tigecycline are the most active agents against MRSA. Based on our monitoring results, erythromycin is not recommended for treating respiratory infections, bacteraemia, or abscesses in children. Intravenous vancomycin is recommended for the treatment of children with invasive MRSA infections. It has been reported that MRSA strains outside of China have retained high susceptibility to clindamycin (Aglua et al., 2018). Greenberg evaluated the effectiveness of clindamycin in infants. They found that 76% of the infants who had MRSA bacteraemia cleared the infection after clindamycin treatment (Greenberg et al., 2020). Clindamycin should be considered for inclusion in the initial antibiotic regimen for treating osteomyelitis and septic arthritis because patients whose initial antibiotic regimens included vancomycin had a longer hospitalization compared with those initiated on a treatment regimen of clindamycin without vancomycin (Weiss et al., 2020). However, we found a particularly high rate of clindamycin resistance among bone and joint-derived MRSA isolates (72.73%), and clindamycin is not suitable to be recommended for the empirical treatment of osteomyelitis. Because of the low number of bone and joint-derived isolates in our study, it needs for more studies. The use of antibiotic treatment is a particular concern owing to the limitation that some antibiotic classes are not suitable for use among neonates, e.g., TMP-SMX, rifampin, gentamicin. Vancomycin and linezolid are recommended for use in treating MRSA infections in neonates, and clindamycin may be considered for the treatment of patients with susceptible isolates who have non-invasive MRSA infections. Although the levofloxacin-resistance rate was low compared to other antibiotics, levofloxacin is not recommended for use in children due to serious side effect.

## Conclusions

The most common age group of patients with MRSA infection is less than 3 years old, and newborns are an important group affected by MRSA infections. MRSA isolates had high rates of resistance to erythromycin and clindamycin but low rates of resistance to levofloxacin, TMP-SMX, gentamicin, and rifampin. No isolate was found to be resistant to vancomycin or linezolid. Changes in the antibiotic resistance rates among the MRSA isolates obtained from 2016 to 2021 were observed, with an increase in the levofloxacin- and TMP-SMX-resistance rates and a decrease in the erythromycin-, clindamycin-, tetracycline-, gentamicin-, and rifampin-resistance rates. The clindamycin-resistance rate of bone and joint-derived MRSA isolates was higher than that of isolates derived from other clinical sources. In areas where clindamycin-resistant MRSA strains are a concern, empirical vancomycin therapy is suggested for the treatment of paediatric osteomyelitis, and clindamycin is not recommended for the treatment of osteomyelitis.

## Data availability statement

The original contributions presented in the study are included in the article/supplementary material. Further inquiries can be directed to the corresponding author.

## Ethics statement

The study protocol was approved by the Ethics Committee of the Children’s Hospital of Fudan University (No. (2020)321). The need for Informed Consent was waived by the Ethics Committee of the Children’s Hospital of Fudan University due to the retrospective nature of the study

## Author contributions

All authors have contributed to the manuscript. XW designed and wrote the draft. XW, CW, LH, HX, CJ, YinC, JD, AL, HD, HC, YipC, JY, TZ, QC, JH and YH collected and analyzed the data, and helped with the data interpretation. HY reviewed the manuscript for its intellectual content and revised the entire work. All authors contributed to the article and approved the submitted version.
